# Effects of the Proprioceptive Neuromuscular Facilitation Technique on Scapula Function in Office Workers with Scapula Dyskinesis

**DOI:** 10.3390/medicina57040332

**Published:** 2021-04-01

**Authors:** Myeungsik Hwang, Sangbin Lee, Chaegil Lim

**Affiliations:** 1 Department of Physical Therapy, Bumin Hospital, Seoul 07590, Korea; mshwang7980@naver.com; 2 Department of Physical Therapy, Namseoul University, Cheonan 31020, Korea; 3 Department of Physical Therapy, Gachon University, Incheon 21936, Korea

**Keywords:** scapula dyskinesis, scapulohumeral rhythm, muscle balance, movement control, DASH, PNF

## Abstract

*Background and Objectives*; Proprioceptive neuromuscular facilitation (PNF) are effective in improving and maintaining Range of motion(ROM), increasing muscular strength and power, and increasing athletic performance, especially after exercise. The scapula patterns defined in PNF are activated within the upper extremity patterns and scapula motions together. Proper function of the upper extremities requires both motion and stability of the scapula. The purpose of this study was to compare the effects of scapula stabilization exercise training involving muscle strengthening, muscle balance, and movement control exercises on office workers with scapula dysfunction. *Materials and Methods*: A total of 42 office workers with scapula dyskinesis were recruited and randomly divided into three groups: muscle strengthening exercise group (*n* = 14), muscle balance exercise group (*n* = 14), and movement control exercise group (*n* = 14). The participants underwent 18 sessions (25 min/session, 3 days a week for 6 weeks) of training involving the three types of exercises. *Results*: The measurement outcomes included the scapula index, measured using a digital Vernier caliper; scapula function, evaluated using the Disability of the Arm, Shoulder, and Hand (DASH) outcome questionnaire (pain and performing, work ability, and sports and art activities); and scapulohumeral movements (scapula upward rotation at humeral abduction angles of 0°, 45°, 90°, 135°, and 180°), evaluated using inclinometers. After the exercise intervention, the scapula index (*p* = 0.002), DASH pain and performing score (*p* = 0.000), DASH work ability score (*p* = 0.000), DASH sports and art activity score (*p* = 0.027), and scapulohumeral movements (scapula upward rotation at 0° (*p* = 0.013) and 45° (*p* = 0.043) humeral abduction) showed significantly greater improvements in the movement control group than in the muscle strengthening and muscle balance groups. *Conclusions*: Thus, proprioceptive neuromuscular facilitation can be used as a rehabilitation intervention for scapula position and movement, pain reduction, and functional improvement in office workers with scapula dyskinesis.

## 1. Introduction

The complex structure of the glenohumeral joint confers the shoulder with the most mobility of any major joint in the human body. This characteristic is primarily due to a limited interface between the humerus and the scapula, requiring the presence of a large network of ligaments, tendons, and other connective tissue elements to provide stability and allow functional movement [1]. The function of the shoulder joint is to place the arms and hands in various positions so that they can perform various tasks [2]. Shoulder pain and dysfunction are among the most common orthopedic problems managed by physiotherapists [3], and the prevalence of shoulder joint diseases is high (~70%) compared with that of other musculoskeletal disorders [4].

Appropriate kinematics of the scapula is crucial for optimal shoulder joint function, such as enabling repetitive movements of the hand over the head [5]. The scapular performs several functions contributing to stability and mobility of scapula complex. As well as s base for muscle attachments, appropriate orientation of the scapula optimizes the length-tension relationship of muscles associated with the scapula complex [6]. Serratus anterior and trapezius are the main muscles that optimize scapula position and scapulohumeral rhythm that reduce pain and increase function [7,8]. In addition, by minimizing the movement limit, shoulder joint movements can be performed as maximum function [9].

However, if the scapula stabilizing muscles are weakened or the function of the shoulder is impaired, the normal position and kinematics of the scapula become altered [10]. An abnormal position of the scapula is a sign of changes in the activity of surrounding muscles [11], and an abnormal movement of the scapula interferes with its coordinated movement with the humerus, resulting in loss of scapulohumeral rhythm and increased scapula damage [5]. Voight et al. [10] suggested that if the scapula fails to perform a stabilizing role, the function of the shoulder joint becomes inefficient and the function of the neuromuscular system decreases, resulting in injury to the shoulder joint. Kibler [12] suggested that the scapula plays a key role in shoulder and arm function as a stable base for optimal muscle activation, a congruent socket for ball-and-socket kinematics, and as a transfer link for developed forces in the kinetic chain.

Among the pathological states associated with the scapula, kinetic chain-based scapula dyskinesis is reported in the literature in conjunction with the term “scapula dysfunction” [13,14]. Scapula dyskinesis is an alteration in the normal position or motion of the scapula during coupled scapulohumeral movements [12]. In general, frequent use of the dominant arm can cause ligament laxity and dysfunction due to contracture of the joint and muscle [12]. This dysfunction may result in ineffective energy transfer, placing added stress on the tissues around the shoulder which must compensate for a weak link in the chain [15], thus changing the kinematics of scapula movement and the scapulohumeral rhythm [16].

In 2019, a systematic literature review on scapula dysfunction reported the following as the main causes of scapula dysfunction: reduced rotator cuff muscle strength, weak scapula muscles, reduced range of motion (ROM) of the shoulder and shoulder joints, lesions in soft tissue, inadequate movement of the scapula, and increased risk of shoulder joint impact [17].

As an intervention for scapula dysfunction, Afsun et al. [18] investigated muscle strengthening exercises, muscle balance exercises, and movement control exercises for the scapula. Kibler [19] also stated that a rehabilitation protocol based on the kinetic chain can gradually restore the scapula dynamic stability by strengthening the scapula stabilizing muscles, thus improving muscle activity and muscle balance. Samir et al. [17] stated that muscle strengthening is a possible intervention strategy for improving recovery and preventing shoulder dysfunction.

In a 2020 systematic literature review of clinical trials on scapula dysfunction, Moghadam [18] reported that the main causes of the scapula dyskinesia are muscle weakness, lack of mobility, presence of lesions in the soft tissues, inappropriate movements, increasing the risk of impact and reduction of the rotator cuff strength.

In more than 68% of cases, shoulder dysfunction occurs in relation to scapula position and movement [19]. However, strengthening exercise of the muscles around the scapula alone is considered an insufficient intervention for scapula dyskinesis and for preventing shoulder joint problems. Thus, the purpose of this study was to apply muscle strengthening, muscle balancing, and movement control exercises for scapula muscles in workers with scapula dyskinesis in order to determine which exercise is more effective.

## 2. Materials and Methods

### 2.1. Ethical Approval

The study was conducted according to the guidelines of the Declaration of Helsinki and approved (5 June 2020) by the Institutional Review Board of Gachon University (1044396-202005-HR-096-01). All participants signed a statement of informed consent before beginning the study.

### 2.2. Participants

Participants were recruited at Seoul B Hospital, and a total of 45 workers agreed to participate in the study. A total of 42 workers completed the study (3 workers stop intervention because of personal reasons). The selection of participants was based on a previous study [11] on scapula dyskinesis targeting office workers. The selection criteria for the participants were as follows: a positive result in the scapula dyskinesis test by orthopedic surgeon or physical therapist; asymmetrical scapula position (left vs. right) at rest; age >20 years but <50 years; ability to perform shoulder abduction in neutral position; and presence of pain in the neck, back, and shoulder. The exclusion criteria were as follows: structure scoliosis, both hand user, a participant who has been receiving conservative physical therapy or therapeutic exercise and manual therapy at a medical institution since the last 3 months, a participant who have been consistently doing specific physical activities and specific exercises from 3 months before the intervention, a participant who have undergone surgical treatment on the shoulder joint and upper limb, and a participant with neurological disorders in the neck and upper extremities.

### 2.3. Procedures

This study was designed as a pretest-posttest experiment. Randomization was intended to minimize an order effect. Baseline measurements of abilities were performed prior to randomization. Subsequently, each participant was allocated to 1 of the 3 groups via allocation codes included in consecutively numbered, sealed, opaque envelopes. Simple randomization was conducted using online algorithm (Research Randomizer; http://www.randomizer.org, accessed on 8 June 2020) by a researcher who was not involved in participant recruitment. To ensure masking, protocols and intervention order were not revealed to participants or clinical evaluators. The experimental group was divided into three intervention groups: muscle strengthening exercise group (MSG), which performed exercises for strengthening the muscles around the scapula; muscle balance exercise group (MBG), which performed exercises for balancing the activity ratio of muscles around the scapula; and movement control group (MCG), which performed exercises for controlling the movement of the scapula and upper limbs according to the commonly applied intervention method for scapula stabilization. Before the intervention, all participants were examined for scapula position, scapula movement, and scapula pain and function. After the preliminary examination, exercises were initiated according to the assigned intervention. The MSG performed exercises to strengthen the muscles around the scapula by lifting their arms in a prone position. The MBG performed exercises to balance the muscle activity of the trapezius and serratus anterior muscles in the side lying and standing positions. In the MCG, the physiotherapist controlled the movements of the scapula and upper limbs in the standing and prone positions with the elbows flexed in 90°.

The exercise time was similar among the three groups: three times a week for 6 weeks, 25 min per session, for a total of 18 sessions. The set and repetition were kept the same, the weight setting had no weight for muscle strengthening, and the muscle balance was set at 10 RM, but no more than 2 kg. After the experiment, the differences before and after the intervention within each group and the differences between the groups were compared. All procedures were conducted by a professional physical therapist.

### 2.4. Interventions

#### 2.4.1. Muscle Strengthening Exercise

**Figure 1:**
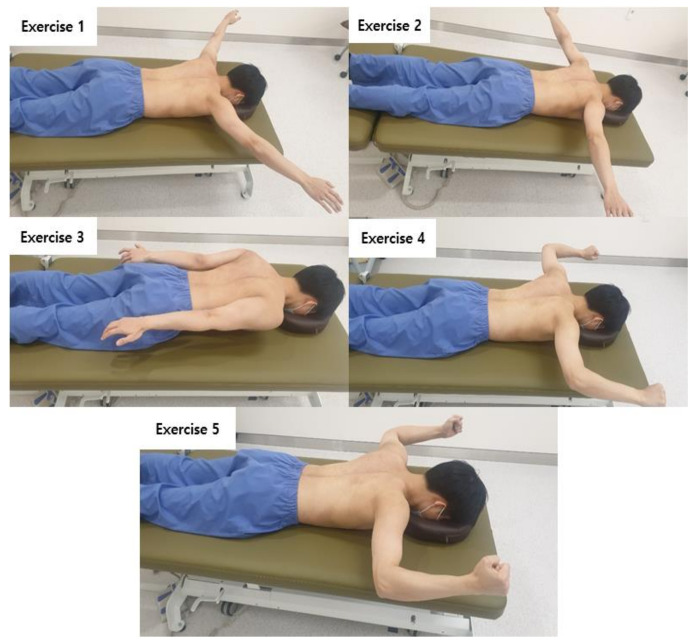
Muscle Strengthening Exercise.

For the muscle strengthening exercise, previous studies by Burkhart et al. [11] and Blackburn [20] were referenced. This exercise is a scapula stabilization exercise that is commonly used in the clinical treatment of scapula dyskinesis [11]. The exercise method consists of five movements and is a bilateral exercise that strengthens the muscles around the scapula by lifting the arm from the prone position (Figure 1).

#### 2.4.2. Muscle Balance Exercise

**Figure 2:**
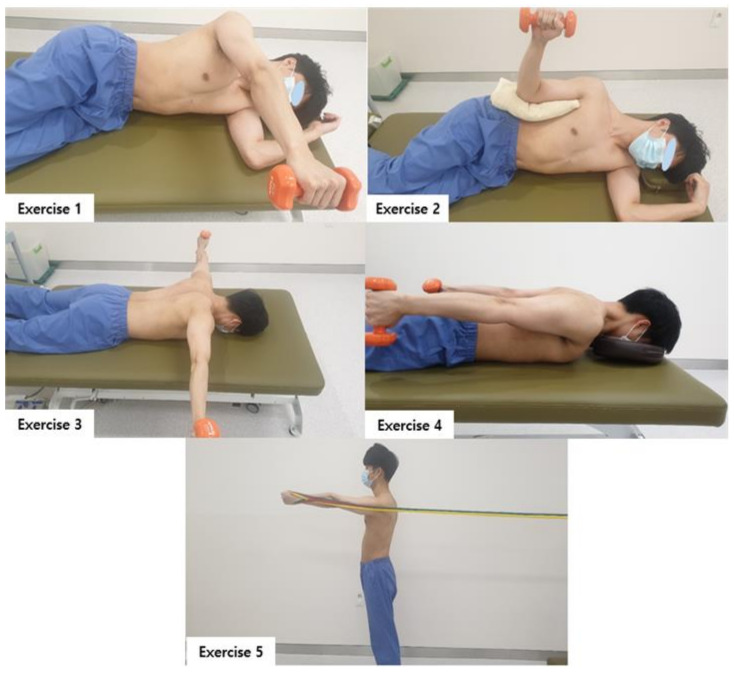
Muscle Balance Exercise.

The muscle balance exercise used in previous studies [21,22,23] was applied for muscle balance exercise of the scapula in this study. This exercise method reduces the hyperactivity of the triceps and balances the activities of the middle trapezius, lower trapezius, and serratus anterior muscles. The exercise was performed for three sets of 10 repetitions, at a 10 repetition maximum load, for a total of 25 min (5 min per set with a 5-min break in between sets). Exercise 1,2,3 is a muscle balance exercise for the upper and middle trapezius, Exercise 1,2,4 is a muscle balance exercise for the upper and lower trapezius, and Exercise 5. It is a muscle balance exercise of the upper trapezius and anterior serratus muscles as a protraction motion [24] (Figure 2). The exercise sequence was carried out as follows by applying a previous study. Week 1: 1, 2, 3, 4, 5/2 weeks: 5, 4, 3, 2, 1/3 weeks: 1, 5, 2, 3, 4/4 weeks: 5, 3, 1, 4, 2/5 weeks: 1, 4, 3, 2, 5/6 weeks: 5, 2, 4, 1, 3.

#### 2.4.3. Movement Control Exercise

For the movement control exercise, the study by Timas et al. [25] was referenced. This exercise is a motor learning method that controls scapula movement by extending the arm forward using the proprioceptive neuromuscular facilitation (PNF) technique. It is an exercise method that dynamically stabilizes the scapula and minimizes shoulder dysfunction. The PNF patterns used in this study were the scapula pattern and the upper limb pattern, and the principles of the “timing for emphasis” and “irradiation” techniques were followed. The movement was controlled by forward flexion of the shoulder joint. This motor learning exercise emphasizes the mobilization of the trapezius and serratus anterior muscles. To dynamically stabilize the scapula when the arm is raised or stretched forward, the upper limb flexion pattern was used. This pattern emphasizes the coordination between the muscles and consists of the D1 (flexion, adduction, external rotation) and D2 (flexion, abduction, and external rotation) components. The PNF scapula pattern is a movement control that improves the dynamic stabilization of the scapula in patients with shoulder joint dysfunction [25]. The PNF technique was performed with “normal timing” for the entire pattern and with “timing for emphasis” emphasizing the scapula position.

### 2.5. Outcome Measurements

#### 2.5.1. Scapula Position

The scapula index (SI) was used to evaluate the scapula position. The SI is an indicator of the general position of the scapula in a static pose and is highly related to the shortening of the pectoralis minor muscle. The SI value was calculated using the following equation: SI = [(distance from the sternal notch to the coracoid process/distance from the posterolateral angle of the acromion to the third spinous process of the thoracic spine) × 100] [26]. Intraclass correlation coefficient (ICC) of SI were intrarater reliabilities = 0.75 and interrater reliabilities = 0.77 [27].

A digital Vernier caliper (Mitutoyo 500-182-30 digital caliper, Kawasaki, Japan) was used to measure the position of the scapula (SI). A smaller SI value indicated shoulder internal rotation, scapula protraction, and abduction, and a larger SI value indicated shoulder external rotation, scapula retraction, and adduction [26].

#### 2.5.2. Scapula Movement

Scapula movement was evaluated by measuring the scapula upward rotation angle at different humeral abduction angles (scapulohumeral rhythm). Two general gravity-based inclinometers (gravity reference inclinometers, HG0020393; OEM, China) were used as the measurement tools. One was attached to the humeral lateral epicondyle shaft, and the other was brought into contact with the scapula spine. This method uses modified gravity-based inclinometers to measure scapula movement in individuals with scapula dysfunction [21]. The starting position was the elbow extension and wrist neutral position in the standing pose, and the upward rotation angle of the scapula spine was measured at shoulder abduction angles of 0°, 45°, 90°, 135°, and 180° [22,23]. The ICC of scapula movement was 0.88 [28].

#### 2.5.3. Disability of the Arm, Shoulder, and Hand Outcome Questionnaire

To evaluate the symptoms and disability of the scapula, a disability outcome questionnaire for the upper limb (Disability of the Arm, Shoulder, and Hand [DASH]; American Academy of Orthopedic Surgeons, Upper Extremity Collaborative Group, Rosemont, IL, USA) was used.

The DASH evaluation tool consists of the following competency evaluations: (1) pain and performing ability evaluation (30 items), (2) work ability evaluation (4 items), and (3) sports and art activity ability evaluation (4 items), with a total of 38 items. Each question was scored on a five-point scale (no difficulty, slightly difficult, moderately difficult, very difficult, or not at all). The score calculation formulas for the three competencies were as follows:

Pain and performing ability = [(sum of scores answered) − 1] ÷ N × 25, where N is the number of questions answered.(1)

Work ability = [{(sum of scores answered) ÷ 4} − 1] × 25.(2)

Sports and art activity ability = [{(sum of scores answered) ÷ 4} − 1] × 25(3)

The lower the evaluation score for each component of DASH, the less pain and the less disability in the evaluated competency [29].

### 2.6. Sample Size Estimation

G power 3.0.1 software (Heinrich Heine University Düsseldorf, Düsseldorf, Germany) was used to determine the sample size. A total of 30 participants were estimated to be required with an effect size of f = 0.50, significance level of 0.05, and power of 0.80. The correlation among representative measures was r = 0.50 when a clinically significant interaction was observed between the two time points (two events: pre and post) and among the three groups.

### 2.7. Statistical Analysis

Statistical analysis was performed using SPSS software (version 23.0; IBM, Armonk, NY, USA). All measured values are presented as means and standard deviations. To verify the normal distribution of the data, all data were tested using the Kolmogorov-Smirnov test. Descriptive statistical analysis and analysis of variance (ANOVA) were used to assess the general characteristics and to test for homogeneity among the participants. A paired *t*-test was performed to analyze differences between the dependent variables according to the measurement period (before and after the experiment), while multivariate ANOVA was used to analyze the differences according to the intervention method (three exercise types). Statistically significant differences were identified using Tukey’s honestly significant difference. Moreover, a partial eta squared (η^2^) was used to explore the effect size. The significance level was set to α = 0.05.

## 3. Results

A total of 42 workers participated in this study (17 men and 25 women). No significant differences were found between the groups in the homogeneity test (*p* > 0.05). The general characteristics of the participants are listed in Table 1.

### 3.1. Scapula Position

#### Scapula Index (SI)

In the MSG, no significant interaction was found in the SI between pre- and post-intervention (*p* = 0.956, Table 2). However, the MBG and MCG showed significantly increased SI after 6 weeks of intervention (*p* = 0.024 and *p* = 0.000, respectively; Table 2), with a statistically significant difference between groups (MCG vs. MSG, *p* = 0.001; Table 2).

### 3.2. Scapulohumeral Movements (Scapula Upward Rotation at Humeral Abduction)

#### Scapula Upward Rotation at 0°–180° Humeral Abduction

No significant interaction was found in the scapula upward rotation at 0°, 45°, 90°, 135°, and 180° (*p* = 0.057, *p* = 0.196, *p* = 0.348, *p* = 0.397, and *p* = 0.617, respectively) humeral abduction of the MSG between pre- and post-intervention (Table 3). However, a significant interaction was found in the scapula upward rotation at 0° (MBG, *p* = 0.018) and 0° and 45° (MCG, *p* = 0.000 and *p* = 0.000, respectively; Table 3) between pre- and post-intervention. A statistical difference was found between the groups in scapula upward rotation at 0° and 45° (MCG vs. MSG and MBG, *p* = 0.010 and *p* = 0.000, respectively; MCG vs. MBG, *p* = 0.046; Effect size = 0.201(0°) & 0.149(45°), Table 3).

### 3.3. DASH Outcome Questionnaire

#### 3.3.1. Pain and Performing Ability

No significant interaction was found in the DASH disability/symptom score of the MSG and MBG between pre- and post-intervention (*p* = 0.650 and *p* = 0.550, respectively; Table 4). The MCG showed significantly increased score after 6 weeks of intervention (*p* = 0.000), and a statistical difference was found between the groups (MCG vs. MSG and MBG, *p* = 0.002 and *p* = 0.000, respectively; Effect size = 0.452, Table 4).

#### 3.3.2. Work Ability

No significant interaction was found in the DASH work ability scores of the MSG and MBG between pre- and post-intervention (*p* = 0.082 and *p* = 0.189, respectively; Table 4). The MCG showed significantly increased score after six weeks of intervention (*p* = 0.001), and a statistical difference was found between the groups (MCG vs. MSG and MBG, *p* = 0.000 and *p* = 0.000, respectively; Effect size = 0.388, Table 4).

#### 3.3.3. Sports & Art Activity Ability

No significant interaction was found in the DASH sports & art activity score of the MSG and MBG between pre- and post-intervention (*p* = 0.082 and *p* = 0.139, respectively; Table 4). The MCG showed significantly increased score after six weeks of intervention (*p* = 0.008), and a statistically significant difference was found between groups (MCG vs. MSG, *p* = 0.036; Effect size = 0.168, Table 4).

## 4. Discussion

Scapula dyskinesis occurs along with imbalance in the scapula muscles, such as hyperactivity in the upper trapezius and hypoactivity in the lower trapezius and serratus anterior [30]. In these cases, muscle strengthening exercises are generally conducted to improve muscle stabilization. The muscle strengthening exercise performed in this study is a stabilization exercise of the scapula and shoulder joints, as reported by Blackburn et al. in 1990 [20]. Blackburn’s scapula stabilization exercise is commonly used by physiotherapists, and it has been reported that there is a significant difference [31] before and after the exercise in terms of muscle strength improvement of the rotator cuff in shoulder joints. However, as scapula dyskinesis is a disorder involving changes in the entire shoulder complex and all shoulder lesions, muscle strengthening of the rotator cuff is not adequately efficient [17]. Therefore, movement control exercises should be conducted considering kinematic aspects rather than unconditional muscle strengthening exercises.

Meanwhile, the serratus anterior plays an important role in stabilizing the scapula, maintaining the thoracic alignment in harmony with the lower trapezius, and dynamically stabilizing the scapula movement [32]. In particular, selective activation of the lower fibers of the serratus anterior has a higher synergy activation with the lower trapezius than with the upper trapezius [33]. If the activation of these muscles is not balanced, the other muscles of the shoulder complex tend to compensate for the movement of the scapula. Therefore, when performing therapeutic exercises for scapula dyskinesis, muscle balance exercises that focus on coordination and activation of the serratus anterior and lower trapezius are efficient [23,27,28].

In this study, the muscle strengthening exercise did not produce significant differences before and after intervention, whereas the muscle balance exercise resulted in significant differences before and after intervention in scapula position and scapula upward rotation at 0° and 45° humeral abduction. In comparison, the movement control exercise produced significant differences in pain and ability, as well as in the position and movement of the scapula. Particularly, in the comparison between groups, the MCG showed a more significant effect than the other groups.

Belling and Jfirgensen [34] stated that scapula movement control exercises would need to be repeatedly performed to return the shoulder joint to a functional position and to consequently stimulate the motor programming of the shoulder complex. This is consistent with the results of the present study, in which movement control exercise caused significant changes in the position and function of the scapula. In addition, Roy et al. [35] reported results similar to our study findings, as they observed that movement control exercises improved movement disorders affecting the scapulothoracic and scapulohumeral areas. The PNF muscle control exercise used in this study differed from the other two exercises in that it involved manual contact by a physiotherapist. This contact helped the participants move in the right direction through the information transmitted to the skin receptors. This also caused contraction and irritation of the co-coordination muscles, thus strengthening the movements, promoting stabilization of the axis, and further strengthening the contraction force of pressure on the muscles. Therefore, it was considered that these effects were related to the more effective results in the MCG than in the other groups. Furthermore, movement control exercise for the scapula produced significant improvements in scapula position, movement, pain, and abilities. In general, if the scapula has an asymmetric position, functional disorder and injury of the upper extremity occur during the arm movements, transferring the proximal energy to the distal regions [16]. An asymmetric scapula position reduces the stabilization ability of the scapula. Moreover, repeated use of strong forces causes soft tissue damage in the upper extremities, while the demand for energy in distal regions increases through the movement of the upper extremities in daily life [36].

This study had some limitations. First, when the position and movement of the scapula is improved, the function is improved and the disability is decreased, but the study did not clearly clarify the mechanism of the interrelationship between the variables. Second, research on the relationship between shoulder motion, muscle strength and symptoms was insufficient. Third, intervention methods were not provided by subdividing scapula dyskinesis type. Therefore, it will be helpful in clinical intervention if subdivided intervention methods are applied for each type and EMG evaluation is added in the future.

On the basis of the results of this study, repeated use of strong forces, such as muscle strengthening exercises, in situations in which the scapula position is not optimal may cause problems in the upper extremities and shoulder joints. Therefore, for an effective treatment, it is important to first stabilize the position of the shoulder bones and to exercise after the normal kinematics of the shoulder joints have been restored.

## 5. Conclusions

In this study, muscle strengthening exercise performed by participants with scapula dyskinesis caused no significant differences in scapula position, movement, pain, and abilities between before and after the intervention. In the group that performed the muscle balance exercise, significant differences were observed in scapula position and movement (0° and 45°) before and after the intervention. In the group that performed movement control exercise, the changes in scapula position, pain, ability and movements (0° and 45°) showed significant differences between before and after the intervention. In addition, movement control exercise was significantly effective than the other two exercises. Thus, as a treatment for scapula dyskinesis, PNF scapula and upper limb movement control exercises are more effective than muscle strengthening and muscle balance exercises.

## Figures and Tables

**Table 1:** General characteristics of the participants (*N* = 42).

	MSG (*n* = 14)	MBG (*n* = 14)	MCG (*n* = 14)	*p*
Sex (male/female), *n* (%)	4/9 (35.7%/64.3%)	4/10 (40.0%/60.0%)	8/6 (57.1%/42.9%)	0.292
Affected side (left/right), *n*	7/7	9/5	7/7	0.697
Age (years)	32.86 ± 6.49	32.01 ± 5.87	32.14 ± 6.66	0.930
Height (cm)	166.05 ± 9.46	165.42 ± 8.92	168.29 ± 9.02	0.685
Weight (kg)	61.83 ± 12.74	62.67 ± 13.97	67.28 ± 13.26	0.514
BMI (kg/m^2^)	22.17 ± 2.45	22.65 ± 3.03	23.61 ± 3.43	0.443

Values are expressed as mean ± standard deviation. Abbreviations: MSG, muscle strength group; MBG, muscle balance group; MCG, movement control group; BMI, body mass index.

**Table 2:** Comparison of scapula position (scapula index) (*N* = 42).

Scapula Position		MSG (*n* = 14)	MBG (*n* = 14)	MCG (*n* = 14)	*p*	E(η^2^)
Scapula index (score)	Pre	68.84 ± 7.66	63.89 ± 5.99	63.86 ± 6.91		
	Post	68.72 ± 7.45	67.40 ± 5.43	72.58 ± 4.83		
	DIFF	0.11 ± 7.79 ^††^	3.50 ± 5.12	8.71 ± 4.79	0.002	0.279
	*p*	0.956	0.024 *	0.000 ***		

Abbreviations: MSG, muscle strength group; MBG, muscle balance group; MCG, movement control group; DIFF, difference; E, effect size; * *p* < 0.05, *** *p* < 0.001, significant intragroup differences; ^††^ *p* < 0.01, significant difference compared with MCG.

**Table 3:** Comparison of scapula upward rotation at different humeral abduction angles (*N* = 42).

Humeral Abduction		MSG (*n* = 14)	MBG (*n* = 14)	MCG (*n* = 14)	*p*	E(η^2^)
0°	Pre	−6.29 ± 3.66	−3.43 ± 5.34	−2.57 ± 3.56		
	Post	−5.64 ± 3.47	−0.14 ± 4.72	1.43 ± 3.79		
	DIFF	−0.64 ± 1.15 ^†^	3.28 ± 4.53 ^†††^	4.00 ± 1.30	0.013	0.201
	*p*	0.057	0.018 *	0.000 ***		
45°	Pre	5.87 ± 3.37	5.21 ± 6.78	4.93 ± 3.14		
	Post	6.50 ± 2.87	5.14 ± 5.48	6.75 ± 3.77		
	DIFF	0.63 ± 1.72	0.07 ± 6.15 ^†^	3.42 ± 1.01	0.043	0.149
	*p*	0.196	0.966	0.000 ***		
90°	Pre	14.57 ± 3.75	17.29 ± 8.10	18.09 ± 5.11		
	Post	15.21 ± 3.64	19.07 ± 6.83	19.07 ± 5.63		
	DIFF	−0.64 ± 2.46	−1.78 ± 9.79	0.97 ± 4.54	0.718	0.017
	*p*	0.370	0.199	0.435		
135°	Pre	34.89 ± 7.82	33.35 ± 13.58	32.70 ± 8.99		
	Post	31.74 ± 9.24	34.78 ± 11.76	34.57 ± 6.61		
	DIFF	3.17 ± 13.58	1.42 ± 12.58	1.87 ± 8.14	0.473	0.038
	*p*	0.397	0.678	0.405		
180°	Pre	43.63 ± 8.38	43.28 ± 15.77	43.40 ± 7.60		
	Post	41.64 ± 11.38	46.57 ± 12.85	44.93 ± 5.58		
	DIFF	1.99 ± 14.56	3.28 ± 15.51	1.52 ± 7.16	0.553	0.030
	*p*	0.617	0.442	0.441		

Abbreviations: MSG, muscle strength group; MBG, muscle balance group; MCG, movement control group; DIFF, difference; E, effect size; * *p* < 0.05, *** *p* < 0.001, significant intragroup differences; ^†^ *p* < 0.05, significant difference compared with MCG; ^†††^ *p* < 0.001, significant difference compared with MCG.

**Table 4:** Comparison of DASH scores (*N* = 42).

DASH		MSG (*n* = 14)	MBG (*n* = 14)	MCG (*n* = 14)	*p*	E(η^2^)
Pain & performing ability (score)	Pre	25.94 ± 16.57	21.08 ± 9.90	27.25 ± 14.40		
	Post	21.73 ± 14.88	20.00 ± 9.95	9.92 ± 8.11		
	DIFF	−4.21 ± 7.80 ^††^	−1.08 ± 1.90 ^†††^	−17.32 ± 12.28	0.000	0.452
	*p*	0.65	0.55	0.000 ***		
Work ability (score)	Pre	22.76 ± 16.73	15.62 ± 9.09	21.42 ± 21.31		
	Post	21.42 ± 15.25	14.28 ± 9.31	10.71 ± 11.15		
	DIFF	−1.34 ± 2.66 ^†††^	−1.33 ± 3.61 ^†††^	−10.71 ± 8.98	0.000	0.388
	*p*	0.082	0.189	0.001 ***		
Sports & art activities (score)	Pre	29.01 ± 18.44	27.23 ± 7.19	24.10 ± 16.04		
	Post	27.67 ± 16.57	24.55 ± 7.13	15.16 ± 14.23		
	DIFF	−1.33 ± 2.66 ^†^	−2.67 ± 6.35	−8.94 ± 10.61	0.027	0.168
	*p*	0.082	0.139	0.008 **		

Abbreviations: MSG, muscle strength group; MBG, muscle balance group; MCG, movement control group; DIFF, difference; E, effect size; ** *p* < 0.01, *** *p* < 0.001, significant intragroup differences; ^†^ *p* < 0.05, significant difference compared with MCG; ^††^ *p* < 0.01, significant difference compared with MCG; ^†††^ *p* < 0.001 significant difference compared with MCG.

## Data Availability

This study is available on request from the corresponding author. The data are not publicly available due to privacy or ethical restrictions.
